# Transactions Alabama Dental Association

**Published:** 1884-07

**Authors:** 


Adjourned.

### Transactions of the Alabama Dental Association.

The Fifth Annual Meeting of the Alabama Dental Association met in Birmingham, April 8th; was called to order at 2:30 p.m. Dr. E. S. Chisholm, of Tuscaloosa, President, and E. Wagner, of Montgomery, Secretary. There were twenty members and ten visitors at the first session.

Dr. Chisholm delivered the President's annual address, and was listened to with great attention.

Drs. Dubois, Allen, and Johnston were appointed a Committee on Membership. Upon the recommendation of the committee the following new members were then elected: F. G. McElhaney, of Auburn, J. G. Turney, of Hartsell, and W. M. Horton and S. W. Talley, of Birmingham.

Annual dues were collected, and the report of the officers received and adopted.

A resolution was adopted extending an invitation to the members of the medical profession to be present at the session; also to the press of the city.

The Publishing Committee presented their report, which was approved.

Adjourned until 9 o'clock to-morrow morning.

### SECOND DAY—Morning Session.

The Association convened at 9 o'clock with several additional members present and several visitors.

The following gentlemen were before the State Board of Dental Examiners, and having passed a satisfactory examination, were licensed to practice, namely: J. E. Frazier, of Springville; B. H. Whittington and Stephen Blaum, of Greenville; R. B. Chapman, of Leon; F. E. Perkins, of Springville; B. B. Seals, of Coatopa.

The reports of Standing Committees were now presented and read. Voluntary papers were now called for.

Dr. W. D. Dunlap, of Selma, read a paper on "Dental Conservatism," which received close attention. It was discussed by Drs. Merrill, McWilliams, Turney, Ball, Morgan, Johnston, and Allen.

Dr. T. M. Allen, of Eufala, read a paper on "Operative Dentistry," which elicited considerable discussion by Drs. Morgan, McWilliams, Turney, and Ball. Dr. Morgan, in his remarks, gave several interesting cases of permanent teeth being lost by improper attention and by tardiness in removing the deciduous teeth.

Dr. George Eubank read an excellent paper on "Dental Education," which was discussed by several members.

"We hope to give these papers in the future number of the Register.—Ed."

An invitation was received to visit the Pratt Coal Mines. This was accepted, and visit made the next morning.

Adjourned.

### Afternoon Session.

The Association was called to order at 3 o'clock. After miscellaneous business, a paper was read by Dr. G. Eubank on

"Some of the Opportunities, Responsibilities, and Encouragements of Life;" this opened a wide field for discussion, which the members improved in an entertaining manner.

Following this was a paper read by Dr. Chisholm on "Mechanical Dentistry." The paper evinced considerable attainments and correct ideas for one who considers himself little more than a student.

A general discussion then took place on "Mechanical Dentistry," in which a number of new and interesting points were made.

Adjourned.

### THIRD DAY--Morning Session.

The whole morning was occupied in the visit to the Pratt Coal Mines; where the members found much to interest them. The descent of between two and three hundred feet, and then passing out quite long distances in the chambers, was a new experience to many of the members. They were afforded special opportunity for observation by the proprietors of the mine; to whom they gave a hearty vote of thanks. They arrived at the hotel prepared to do ample justice to the tables of the host.

The forenoon was devoted to clinics, in which a number of the members were engaged.

Dr. Allen, of Eufaula, illustrated the use of the electric mallet and electric engine, to the admiration of all who witnessed it.

Demonstrations were given in modes of using different materials.

Quite a large number of fine microscopic specimens, embracing sections of teeth, bones, and their tissues, were exhibited by Dr. G. Eubank, many of them prepared by himself.

Adjourned.

### FOURTH DAY—Morning Session.

The Society met at the usual hour, President Chisholm in the chair. The Board of Dental Examiners, acting under the law of regulating the practice of dentistry in Alabama, made a report, in which they recommended several changes in the law, regulating the practice of dentistry. The most important of these is, that the law governing the granting of certificates by the State Dental Board be changed, so as to conform to the law concerning certificates where applicants, upon examination, fall short of the standard. That will insure capability in the profession. The law at present requires the Board to grant certificates to applicants without examination who present diplomas.

Upon this there was some discusssion, but there was a very general concurrence in the recommendation of the Board.

The following were elected officers of the Association for the ensuing year:

President, W. R. McWilliams, Athens; First Vice-President, George Eubank, Birmingham; Second Vice-President, E. Wagner, Montgomery; Secretary, C. A. Merrill, Birmingham; Treasurer, G. M. Rousseau, Montgomery.

The next annual meeting is to be held at Montgomery on the second Tuesday in April, 1885.

The convention adjourned and most of the members left for home on the afternoon and night trains.

